# Design of Versatile Top‐Down Transfer by Thermal Release Tape/Poly(methyl methacrylate) (TRT/PMMA) Bi‐Supporting Layers Toward All‐Transfer Transition Metal Dichalcogenide Material Based Transistor Arrays

**DOI:** 10.1002/smsc.202300144

**Published:** 2023-12-21

**Authors:** Ying-Chun Shen, Bang-Kai Wu, Tsung-Shun Tsai, Mingjin Liu, Jyun-Hong Chen, Tzu-Yi Yang, Ruei-Hong Cyu, Chieh-Ting Chen, Yu-Chieh Hsu, Chai-Hung Luo, Yu-Qi Huang, Yu-Ren Peng, Chang-Hong Shen, Yen-Fu Lin, Po-Wen Chiu, Ya-Chin King, Yu-Lun Chueh

**Affiliations:** ^1^ Department of Materials Science and Engineering National Tsing-Hua University Hsinchu 30013 Taiwan; ^2^ Institute of Electronics Engineering National Tsing Hua University Hsinchu 30013 Taiwan; ^3^ College of Semiconductor Research National Tsing-Hua University Hsinchu 30013 Taiwan; ^4^ Department of Physics National Sun Yat-Sen University Kaohsiung 80424 Taiwan; ^5^ National Applied Research Laboratories Taiwan Semiconductor Research Institute Hsinchu 30013 Taiwan; ^6^ Department of Physics National Chung Hsing University Taichung 40227 Taiwan

**Keywords:** electrode transfer, large-scale transfers, selective transfer, top-down transfer, transition metal dichalcogenides materials

## Abstract

A top‐down transfer process is developed via a rolling process associated with thermal release tape/poly(methyl methacrylate) (PMMA) bi‐supporting layers to realize large‐scale transfer processes on transition metal dichalcogenide materials. A 2‐inch MoS_2_ thin film transferred on SiO_2_/Si substrates with high integrity and a yield of ≈99% can be successfully achieved via the proposed process. MoS_2_‐based transistors with a transferred Au thin film as the contact electrode indicate a lower contact resistance of 8.4 kΩ with improved mobility and a higher on/off ratio compared with that of the MoS_2_‐based transistors with the evaporated Au thin film as the contact electrode. By applying the difference in adhesion force between metal oxides and metal on MoS_2_ and PMMA surfaces, the selective transfer of MoS_2_ films can be demonstrated. Furthermore, all‐transferred MoS_2_‐based transistor arrays are demonstrated by combining the selectively transferred MoS_2_ film as the channel material and the transferred Au thin films as the contact electrode, which results in uniform electrical properties featuring a carrier mobility of 10.45 cm^2^ V^−1^ s^−1^, a subthreshold swing of 203.94 mV dec^−1^, a normalized *I*_on_ of 8.3 μA μm^−1^, and an on/off ratio of 10^5^.

## Graphical abstract

A top‐down transfer process involving a rolling process using a thermal release tape/poly(methyl methacrylate) bi‐supporting layers is demonstrated to realize all‐transferred MoS_2_‐based transistor arrays. Combining a selectively transferred MoS_2_ channel and transferred Au contact electrodes, the arrays demonstrate uniform electrical properties with a carrier mobility of 10.45 cm^2^ V^−1^ s^−1^, and a normalized *I*_on_ of 8.3 μA μm^−1^.

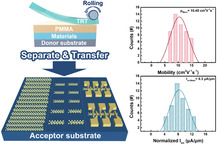

## 1 Introduction

Transition metal dichalcogenide (TMDC) materials are promising candidates for miniaturization to the atomic scale because of their excellent electronic and optical properties.^[^ ^1^ ^]^ In general, high‐performance devices containing TMDC materials require defectless large‐scale TMDC materials and clear metal–TMDC contacts.^[^ ^2^, ^3^, ^4^ ^]^ TMDC materials can be transferred from a donor substrate to an acceptor substrate for integration with existing Si‐based and future technologies. However, transfer‐induced defects, such as wrinkles, cracks, folds, and distortions, are generated in TMDC materials during the transfer processes, thus deteriorating their electrical and structural properties.^[^ ^5^, ^6^, ^7^ ^]^ Therefore, a large‐scale and defectless transfer method must be developed urgently to maintain the quality of TMDC materials.

Transfer methods can be broadly classified into two categories: bottom‐up and top‐down. The bottom‐up transfer method, namely, the wet transfer method, involves transferring TMDC materials from a donor substrate to an acceptor substrate by immersing the acceptor substrate in a liquid, typically water or organic solvents.^[^ ^8^, ^9^ ^]^ An adjustable wettability‐assisted transfer (AWAT) method using a low‐polarity transfer medium to enhance the wettability between the interface of TMDC layers and acceptor substrates has been demonstrated; this method affords TMDC‐based transistors with fewer wrinkles as well as improved mobility and a higher on/off ratio.^[^ ^10^ ^]^ Meanwhile, the bottom‐up transfer process has been widely used because of its simplicity and speed. The top‐down transfer method, also known as the dry transfer method, involves transferring TMDC materials to an acceptor substrate without the use of any liquid.^[^ ^11^, ^12^, ^13^ ^]^ However, transferring large‐scale TMDC materials without damaging TMDC materials is challenging. Yu et al. developed a dry transfer method using poly(dimethylsiloxane) (PDMS) as a supporting layer to transfer a 2‐inch MoS_2_ film.^[^ ^14^ ^]^ Shim et al. demonstrated a layer‐resolved splitting technique using a Ni metal film as a supporting layer, which can isolate two‐dimensional (2D) layered materials at the wafer scale from monolayer to multilayer 2D materials.^[^ ^15^ ^]^ However, this method is challenging because it requires reasonable control over the conditions and pressures on the supporting layer during the transfer process to avoid damage to the materials. In addition to the transfer process, metal contacts contribute significantly to the performance of TMDC‐based devices. However, conventional metal deposition processes such as evaporation and sputtering involving ion bombardment introduce defects, strain, disorder, and metal diffusion into TMDC semiconductors, thus resulting in high contact resistance because of Fermi‐level pinning at the interface between metal contacts and TMDC materials.^[^ ^16^, ^17^, ^18^, ^19^, ^20^ ^]^ This issue can be mitigated by implementing van der Waals (vdW) contacts into TMDC‐based devices. Liu et al. developed MoS_2_ vertical transistors using vdW metal contacts by transferring prefabricated Ag electrodes, thus minimizing the leakage tunneling current.^[^ ^21^ ^]^ Additionally, Liu et al. demonstrated a graphene‐assisted metal transfer printing technique, which can be used to transfer metal electrodes with weak and strong adhesion to form vdW metal–semiconductor interfaces, thus avoiding disorder and Fermi‐level pinning.^[^ ^22^ ^]^

In this study, a top‐down transfer process involving a rolling process using thermal release tape (TRT)/poly(methyl methacrylate) (PMMA) bi‐supporting layers to realize large‐scale transfer processes on TMDC materials (MoS_2_ and WS_2_) and the implementation of vdW contacts using an Au metal film as the electrode to reduce transfer‐induced defects were demonstrated. TRT/PMMA was selected as the bi‐supporting layer to maintain the flatness and integrity of the target materials (i.e., TMDC materials such as MoS_2_ and WS_2_ layers, as well as Au films as the contact electrode) with the assistance of the rolling process. Raman spectra were obtained to investigate the quality of TMDC materials before and after the transfer process. Optical microscopy (OM) and atomic force microscopy (AFM) results were used to investigate the yield and surface morphology of the transferred TMDC films. The result indicates that a 2‐inch MoS_2_ layer can be transferred with a yield exceeding ≈99% and a roughness of less than 0.6 nm. The top‐down transfer process using TRT/PMMA bi‐supporting layers not only transferred large‐scale TMDC layers, but also transferred the Au predeposited film as the contact electrode, thus resulting in a lower contact resistance (*R*_c_) of 8.4 kΩ with improved mobility and a higher on/off ratio compared with that of the MoS_2_‐based transistors with an evaporated Au thin film as the contact electrode. Furthermore, selective transfer was performed on the TMDC layers using the appropriate metals with different metal oxidation layers as barriers; subsequently, the selective transfer mechanism was investigated comprehensively. This versatile dry‐transfer process can be further improved to achieve an all‐transfer TMDC‐based device using transferred MoS_2_ layers as channels and transferred Au films as contact electrodes. The statistical results of the electrical properties show the excellent uniformity of the arrays.

## 2 Results and Discussion

**Figure 1:**
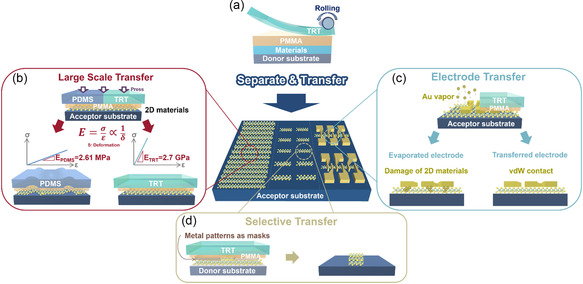
Schematic illustration of the functions by the top‐down transfer processes using TRT/PMMA bi‐supporting layers. a) TRT is attached to PMMA/target material/donor substrate with the help of the rolling process. b–d) Schematic illustrations of large‐scale transfer (b), electrode transfer (c), and selective transfer (d).

**Figure** **1** shows a schematic illustration of the different functionalities of this top‐down transfer process, including large‐scale, electrode, and selective transfer processes. First, a PMMA layer was spin‐coated onto the material/donor substrates. Subsequently, the TRT was stacked on a PMMA/material/donor substrate via rolling, as depicted in Figure 1a. The top‐down transfer process via rolling using the TRT/PMMA bi‐supporting layers, denoted as the top‐down transfer process, can be segmented into three main processes: separation, top‐down transfer, and removal, as shown in Figure S1 (Supporting Information). In the separation step, PMMA was spin‐coated onto the target material as the first supporting layer to protect the integrity of the target material and enhance the adhesion between the TRT and target material. Second, the TRT was placed on the PMMA/target material, followed by a rolling process to eliminate bubbles or air gaps between the TRT and PMMA interface. Third, the TRT/PMMA/target material was separated from the donor substrate using deionized (DI) water. After separation, the TRT/PMMA/target material was evaporated at room temperature to remove the DI water adsorbed on the target material. Consequently, the TRT/PMMA/target material was attached to the target substrate with the assistance of the rolling process, thus allowing the TRT/PMMA/target material stacking layers to be transferred onto the target substrate completely and smoothly. To complete the top‐down transfer process, the TRT/PMMA/target material was forced onto the substrate and simultaneously heated to reduce the adhesiveness of the TRT layer. After removing the TRT, the device was immersed in hot acetone to remove the PMMA, which allowed the target material to be transferred onto an acceptor substrate. Selecting the appropriate supporting layer is crucial for achieving the large‐scale transfer of a TMDC layer with a highly flat surface. The elasticity of the supporting layer affects the flatness of the TMDC layer and thus the transfer (Figure 1b). Generally, the deformation after the top‐down transfer process is related to the elasticity, which can be described by Hooke's law, i.e., $E=\frac{\sigma }{\varepsilon }=\frac{FL}{A\delta }$, where *E*, *σ*, *ε*, *F*, *L*, *A*, and *δ* represent the Young's modulus (i.e., modulus of elasticity), stress, strain, applied force, length, applied area, and deformation, respectively. Therefore, the deformation after the top‐down transfer process decreases when using the TRT/PMMA bi‐supporting layers as the transfer layer, as compared with the conventional top‐down transfer process using PDMS/PMMA as the transfer layer. The correlation between the TRT and PDMS can be referred to as the difference in Young's modulus, i.e., the stiffness of the material. For the PDMS with a low Young's modulus (i.e., 2.61 MPa), the PDMS transfer layer is highly likely to be affected by the strain‐induced mismatch inside the TMDC layers.^[^ ^23^ ^]^ By contrast, the TRT, which has a high Young's modulus (2.7 GPa for the PET layer of the TRT), increases the possibility of maintaining the flatness and integrality of the TMDC layers after the top‐down transfer process.^[^ ^24^ ^]^ Regarding the metal contact issue on the TMDC layers, the conventional metal deposition involving ion bombardment will result in interface damage, such as defects and chemical disorder at the contact region between the metal and TMDC layers, as shown in Figure 1c. Implementing a vdW contact on the TMDC layers has been shown to mitigate this undesired problem. To illustrate this concept, the vdW contact method using TRT/PMMA bi‐supporting layers was used to transfer the predeposited electrodes on top of the TMDC layers, which eliminated interface damage and Fermi‐level pinning. The selective transfer of TDMC layers by the TRT/PMM bi‐supporting layers afforded the selective transfer of TMDC layers via metal layers as a barrier, as shown in Figure 1d. By selecting the appropriate metals, the TRT/PMMA bi‐supporting layers can remove the TMDC layer unshielded by the metal region instead of all the regions of the TMDC layers.

**Figure 2:**
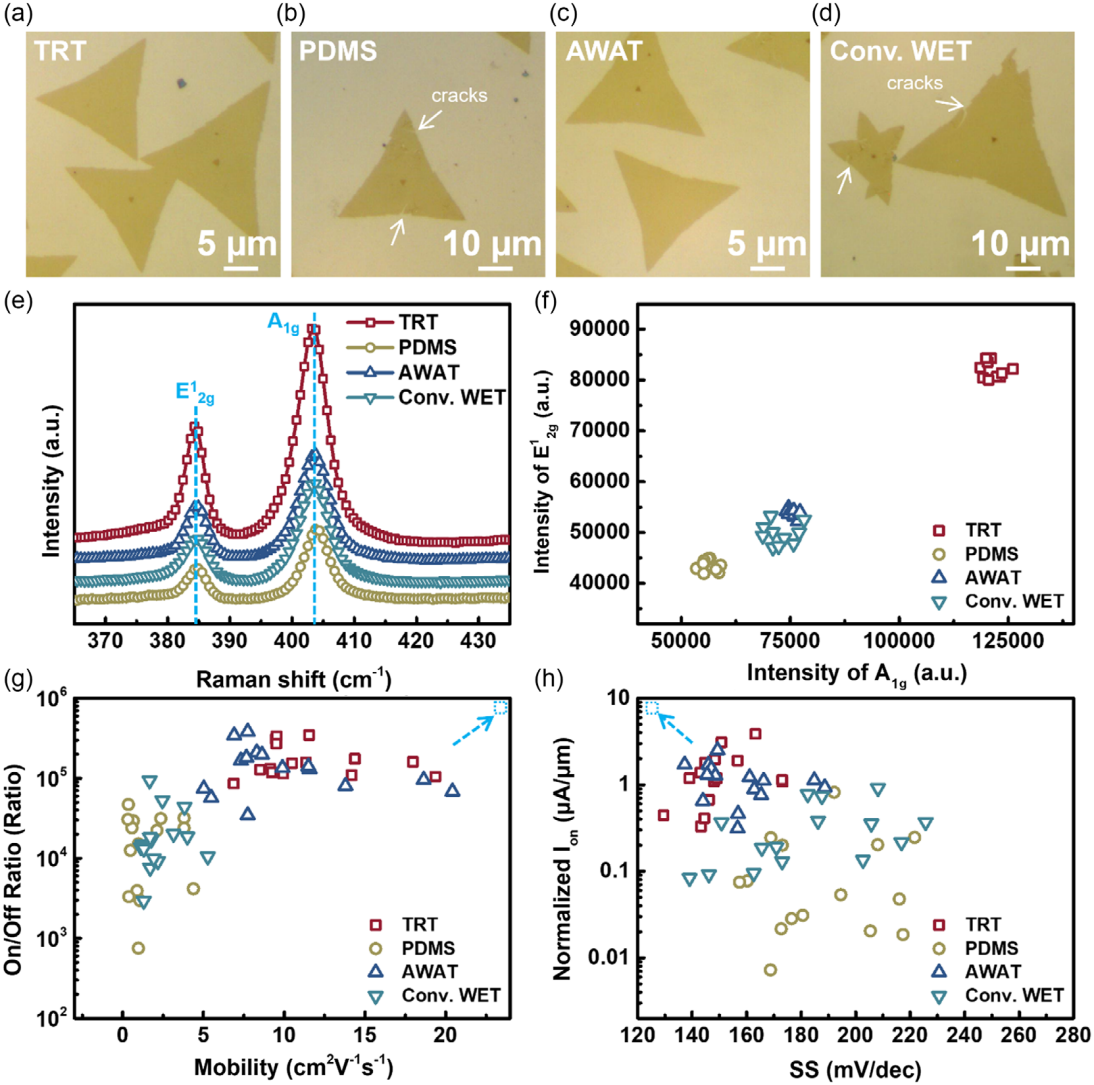
Optical and electrical results of MoS_2_ flakes transferred by four methods. a–c) OM images of MoS_2_ flakes transferred by TRT/PMMA bi‐supporting layers (a), PDMS/PMMA bi‐supporting layers (b), conventional wet transfer (c), and AWAT (d). e) Raman spectra and f) statistical results of E^1^_2g_ and A_1g_ peak intensities of MoS_2_ layers transferred by four methods. g) On/off ratio versus mobility and h) normalized *I*_on_ versus SS of the MoS_2_‐transistors underwent different transfer processes.

To investigate the advantages of the top‐down transfer process, four different transfer methods, i.e., TRT, PDMS,^[^ ^14^ ^]^ AWAT,^[^ ^10^ ^]^ and conventional WET,^[^ ^8^ ^]^ were compared, as shown in **Figure** **2**. Figure 2a–d shows the corresponding OM images of MoS_2_ flakes transferred via TRT/PMMA, PDMS/PMMA, AWAT, and conventional WET transfer processes, respectively. A comparison of the top‐down transfer processes of the TRT/PMMA bi‐supporting layers with those of the PDMS/PMMA bi‐supporting layers showed that highly dense cracks were formed in the MoS_2_ flakes by the PDMS/PMMA bi‐supporting layers owing to the low Young's modulus of PDMS. Regarding the bottom‐up transfer processes of AWAT and conventional WET, the MoS_2_ flakes were distorted and cracked after transfer via conventional WET etching because of the low wettability of DI water. Raman spectra were obtained before and after all transfer processes, as shown in Figure S2a,e,f (Supporting Information). Figure S2a (Supporting Information) shows the Raman spectra of MoS_2_ flakes on sapphire substrates before the top‐down transfer processes and the corresponding intensities of E^1^_2g_ and A_1g_ obtained from 10 MoS_2_ flakes before the transfer process using four different transfer methods. The similar peak positions and intensities of the MoS_2_ flakes indicate that MoS_2_ flakes with similar uniformity and quality can be achieved before the transfer process using the four different transfer methods. Figure 2e shows the Raman spectra of MoS_2_ flakes on SiO_2_/Si substrates after the transfer process using four different transfer methods as well as the intensities of E^1^_2g_ and A_1g_ for MoS_2_ flakes after the transfer process, as shown in Figure 2f. The Raman intensities of MoS_2_ on the sapphire substrates (i.e., before transfer processes) were weaker than those on the SiO_2_/Si substrates (i.e., after transfer processes) owing to the transparency of sapphire and the fact that the Raman spectrum was based on light reflected from MoS_2_.^[^ ^25^ ^]^ The peak intensity of the MoS_2_ flakes after the transfer process via the four different transfer methods differed. Additionally, the MoS_2_ flakes transferred via the top‐down transfer process using TRT/PMMA bi‐supporting layers showed the highest intensities for the two peaks. By contrast, the MoS_2_ flakes subjected to other transfer processes showed similar results with a lower intensity distribution, thus indicating that the quality of the MoS_2_ flakes can be maintained by the TRT/PMMA bi‐supporting layers. Furthermore, MoS_2_‐based back‐gate transistors were fabricated to investigate the electrical characteristics of the MoS_2_ flakes after the transfer process using four different transfer methods. The on/off ratio versus mobility trend was obtained using 15 transistors from each transfer process, as shown in Figure 2g. The corresponding *I*_DS_–*V*_GS_ curves after the transfer process using the four different transfer methods are presented in Figure S3 (Supporting Information), which shows that all transistors exhibited n‐type behavior. The transistors after being subjected to the top‐down transfer processes using TRT/PMMA bi‐supporting layers showed the highest on‐state current (*I*_on_), whereas those after being subjected to the top‐down transfer processes using PDMS/PMMA bi‐supporting layers showed the lowest *I*_on_. The carrier mobility (*μ*) can be extracted from the transfer curves and calculated as follows: $\mu =\frac{L}{W\times C\times V_{D}}\times \frac{dI_{D}}{dV_{G}}$, where *L*, *W*, and *C* are the channel length, contact width, and capacitance of the gate oxide, respectively.^[^ ^26^ ^]^ The transistors after the transfer using PDMS/PMMA bi‐supporting layers or conventional WET processes indicated a lower on/off ratio of <10^5^ and a mobility of <5 cm^2^ V^−1^ s^−1^. By contrast, after the transfer using TRT/PMMA supporting layers as well as AWAT, the transistors exhibited a higher on/off ratio of ≈10^5^ and an average mobility of ≈10 cm^2^ V^−1^ s^−1^, which allowed them to pursue the desired corner (the blue square indicated in the upper right of Figure 2g). Figure 2h shows the normalized *I*_on_ as a function of the subthreshold swing (SS) of 15 transistors obtained during each transfer process. The transistors transferred by the PDMS/PMMA bi‐supporting layers or conventional WET showed a large distribution trend, thus indicating the low uniformity of the transistor. Meanwhile, the transistors transferred using TRT/PMMA bi‐supporting layers as well as AWAT resulted in a narrow distribution, a higher normalized *I*_on_ by approximately 1 μA μm^−1^, and a lower SS of less than 200 mV dec^−1^. As such, they were able to achieve the desired corner, which is the blue square indicated in the upper left of Figure 2h. The transfer processes using both the TRT/PMMA bi‐supporting layers and AWAT yielded better electrical characteristics. However, AWAT (i.e., one of the bottom‐up processes) is suitable for small‐area transfers and new materials used in laboratories but not for large‐scale transfers and industry technology and integration.

**Figure 3:**
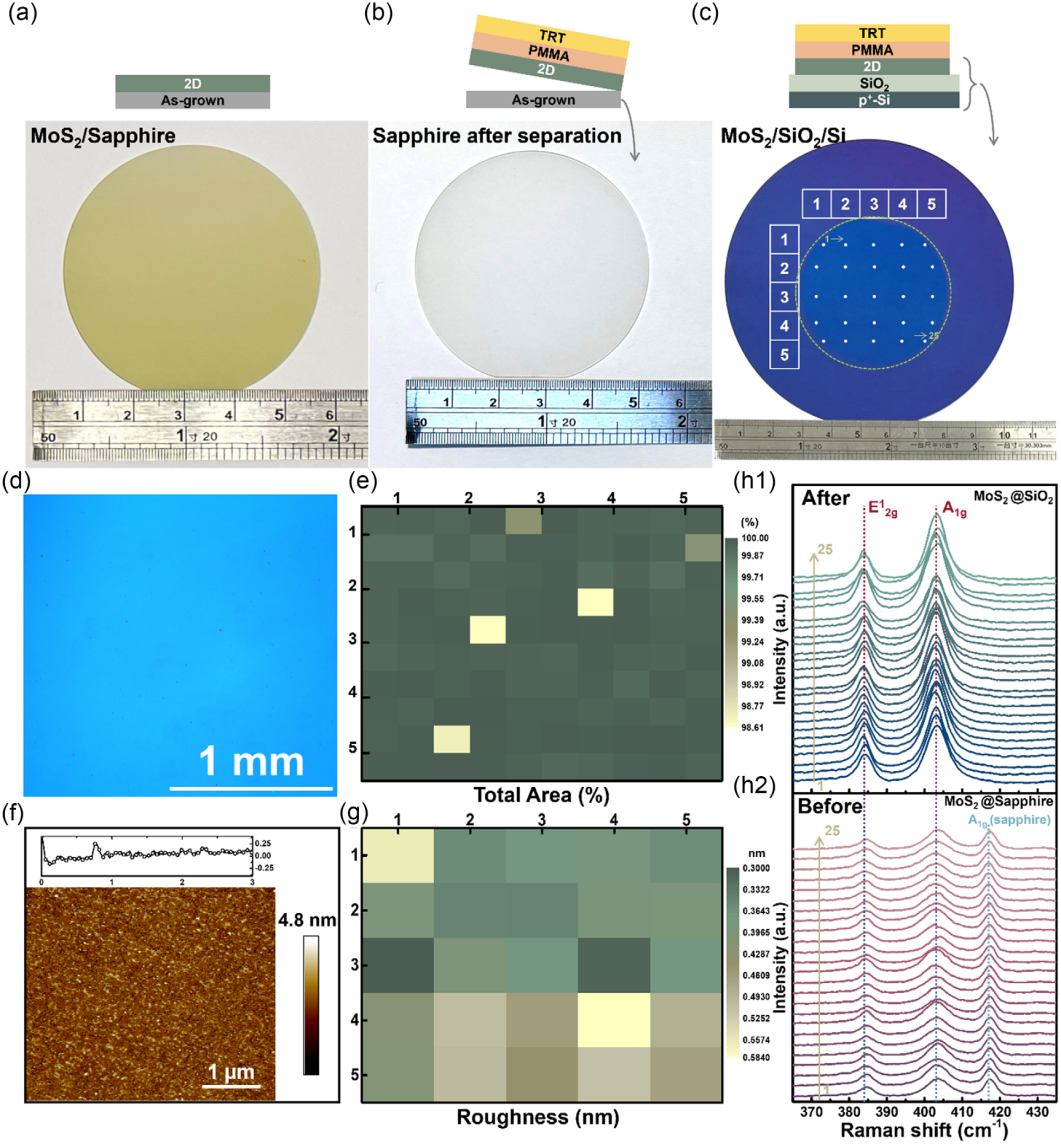
Large‐scale transfer of MoS_2_ layers by TRT/PMMA bi‐supporting layers. a–c) Pictures of as‐grown MoS_2_ layers on a sapphire substrate (a), the sapphire after separating MoS_2_ layers (b), and the MoS_2_ layers transferred on a 4 inch SiO_2_/Si substrate (c). d) OM image of the transferred MoS_2_/SiO_2_. e) The yield of the transferred MoS_2_/SiO_2_ extracted by 100 OM images. f) AFM image of the transferred MoS_2_/SiO_2_. g) The roughness of the transferred MoS_2_/SiO_2_ was extracted by 25 AFM images. h_1_) Raman spectra of the transferred MoS_2_/SiO_2_. h_2_) Raman spectra of as‐grown MoS_2_/sapphire.

To demonstrate the top‐down transfer of large‐scale TMDC with high integrity and flatness, 2‐inch MoS_2_ layers were grown via chemical vapor deposition on a sapphire substrate, followed by a top‐down transfer process onto a 300‐nm SiO_2_/p‐Si substrate using TRT/PMMA bi‐supporting layers, as shown in **Figure** **3**a–c. Figure 3a shows the MoS_2_ layers grown in all regions of the sapphire donor substrate. Figure 3b shows a photograph of the sapphire donor substrate after the MoS_2_ continuous film was peeled off. The cleanliness of the substrate indicated that the MoS_2_ layers can be completely peeled off by the TRT/PMMA supporting layers. Figure 3c shows an image of the MoS_2_ layers after they were transferred onto a SiO_2_/Si acceptor substrate, which indicated that the 2‐inch MoS_2_ layers were successfully and completely transferred (the region within the dashed green circle). To investigate the yield of the transferred large‐scale MoS_2_ layers, OM images were captured, as shown in Figure 3d and S4 (Supporting Information). As shown in Figure 3d, the MoS_2_ layers were completely transferred onto the substrate without any defects at the millimeter scale. Figure S4 shows 100 OM images captured from the transferred 2‐inch MoS_2_ layers, where four OM images were captured on a 1 cm × 1 cm scale. The untransferred regions are shown in red and calculated using ImageJ to investigate the yield of the entire transferred film. As shown in Figure 3e, the yield can exceed ≈99%. Furthermore, to investigate the roughness of the transferred film, AFM was conducted on both the as‐grown and transferred MoS_2_ layers. Figure 3f shows an AFM image of the transferred MoS_2_ continuous film, which shows no defects such as holes, wrinkles, or cracks on the micrometer scale. Figure S5a,b (Supporting Information) shows the AFM images of the SiO_2_ accepted substrate and as‐grown MoS_2_ layers, respectively. The surface roughness values of the SiO_2_ and as‐grown MoS_2_ layers were 0.172 and 0.751 nm, respectively. Figure S6 (Supporting Information) shows 25 AFM images of the transferred MoS_2_ layers corresponding to the white dots in Figure 3c. Based on an analysis of Figure 3g, the roughness of the MoS_2_ layers was less than 0.6 nm, thus indicating the excellent flatness of the transferred MoS_2_ layers. Figure 3h_1_,h_2_ shows the Raman spectra of the MoS_2_ layers before (Figure 3h_2_) and after (Figure 3h_1_) the transfer process. The spectra of the MoS_2_ layers were measured and obtained for each 1 cm × 1 cm area (corresponding to the region marked by white dots in Figure 3c). The 25 Raman spectra of the as‐grown MoS_2_ layers showed identical peak positions and intensities of E^1^_2g_ and A_1g_, thus indicating the high uniformity and homogeneity of the MoS_2_ layers (Figure 3h_2_). Moreover, the 25 Raman spectra of the transferred MoS_2_ layers indicate that the film can retain its quality after the transfer process owing to the slight change in the intensities and positions of the two peaks (Figure 3h_1_).

Furthermore, 5 cm × 5 cm WS_2_ layers were successfully transferred onto a 4 inch, 300 nm SiO_2_/p‐Si wafer via the TRT/PMMA bi‐supporting layers, as shown in Figure S7 (Supporting Information). Figure S7a (Supporting Information) shows an image of a 5 cm × 5 cm WS_2_ layer transferred onto a SiO_2_ substrate (the region within the dashed square). Similarly, the WS_2_ layers were completely transferred onto the substrate with high integrity on the millimeter scale, as shown in Figure S7b (Supporting Information). Figure S8 (Supporting Information) shows 100 OM images obtained from the transferred 5 cm × 5 cm WS_2_ layers, where four OM images were captured on a 1 cm × 1 cm scale. As shown in Figure S7d (Supporting Information), the center of the WS_2_ layers was transferred more successfully, with a yield of ≈99.6%. Although more defects were observed at the edges of the transferred WS_2_ layers, the yield exceeded 99.17%. As shown by the AFM image in Figure S7c (Supporting Information), no significant defect in the micrometer scale was observed on the transferred WS_2_ layers. Figure S9 (Supporting Information) shows the AFM image of the as‐grown WS_2_ layers, in which a roughness of 0.251 nm was measured. Figure S10 (Supporting Information) shows 25 AFM images of the transferred WS_2_ layers, which correspond to the white dots marked in Figure S7a (Supporting Information). Based on an analysis of Figure S7e (Supporting Information), the roughness of the transferred WS_2_ layers was less than 0.63 nm, thereby indicating the superb flatness of the transferred WS_2_ layers. Figure S7f (Supporting Information) shows the Raman spectra of the WS_2_ layers before and after the transfer, which were randomly measured and obtained in four areas of the WS_2_ layers, as shown in Figure S7a (Supporting Information).

**Figure 4:**
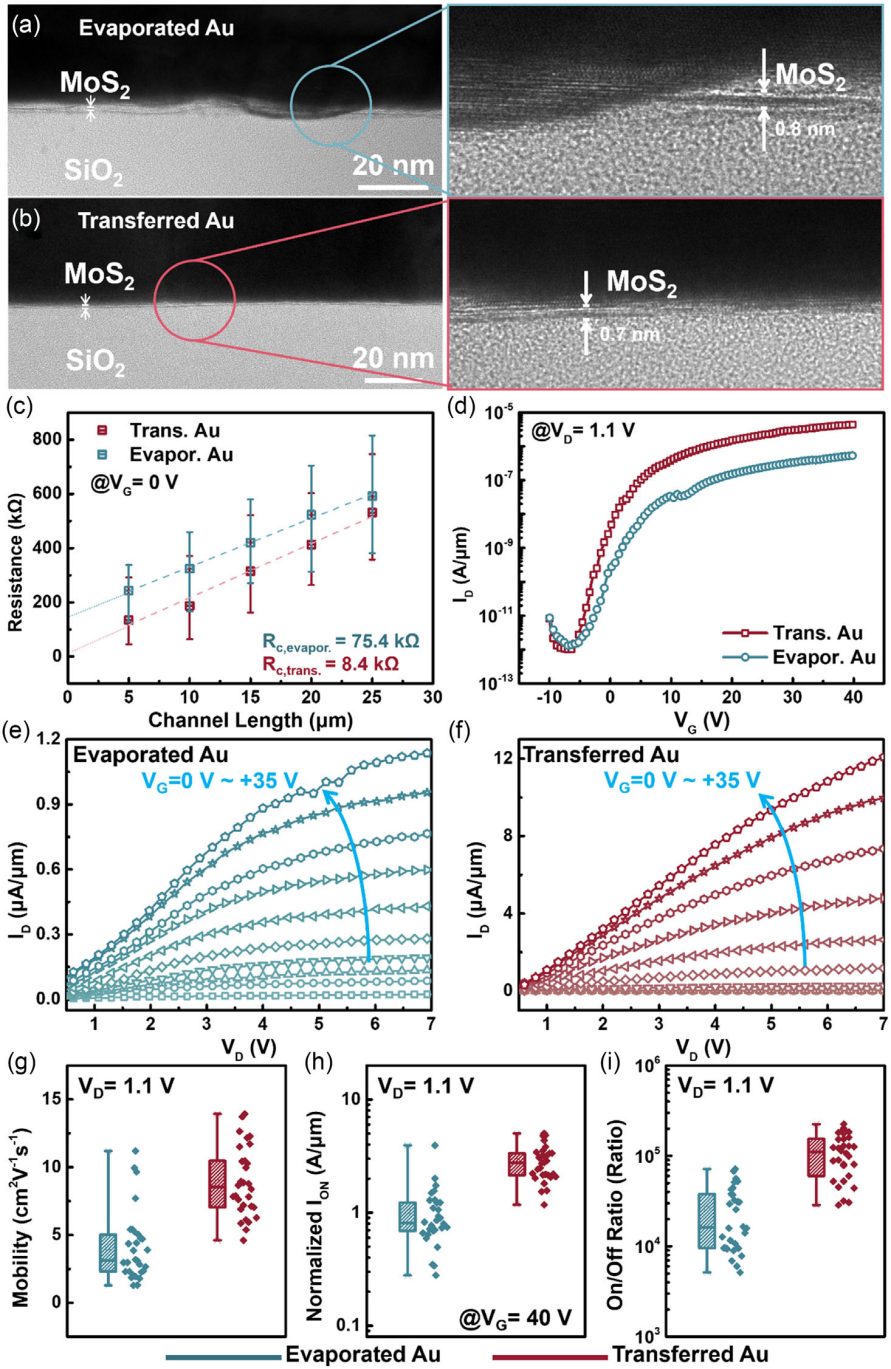
Au electrode transfer by TRT/PMMA bi‐supporting layers. a,b) HRTEM images of evaporated Au thin film onMoS_2_ layers (a) and transferred Au thin film on MoS_2_ layers (b). c) TLM measurements and d) transfer curves of MoS_2_‐based transistors with evaporated and transferred Au thin films as electrodes. e,f) Output curves of MoS_2_‐based transistors with evaporated Au thin film (e) and transferred Au thin film (f) as the electrode. g–i) Statistical results of mobility (g), normalized *I*_on_ (h), and on/off ratios (i) of MoS_2_‐based transistors with evaporated and transferred Au thin film as electrodes.

The metal deposition process involving atom bombardment or high‐energy hot atoms will result in interfacial damage.^[^ ^21^ ^]^ When directly depositing an Au thin film on the SiO_2_ substrate, the Au/SiO_2_ interface was bombarded and damaged; the corresponding cross‐sectional HRTEM images are shown in Figure S11 (Supporting Information). Interestingly, when transferring an Au thin film onto the SiO_2_ substrate, the interface between the Au thin film and SiO_2_ film was smooth and clear, as shown in Figure S12 (Supporting Information). Compared with the SiO_2_ thin film, the TMDC layers were more fragile and thus could be easily damaged during the fabrication of source and drain electrodes. Therefore, transferring the metal electrodes onto the TMDC layers to create a vdW contact is a promising solution for eliminating damage and Fermi‐level pinning. In this study, an Au thin film with a thickness of 50 nm was selected as the electrode owing to its ability to form long‐term air‐stable contacts without requiring doping.^[^ ^27^ ^]^ The detailed transport processes of the Au thin film are shown in Figure S13 (Supporting Information), which involved the initial deposition of an Au thin film as the metal electrode on a donor substrate. Subsequently, the electrode was peeled off and attached to the TMDC layers using TRT/PMMA bi‐supporting layers. **Figure** **4** shows a comparison of MoS_2_‐based transistors with evaporated Au and transferred Au thin films of identical thicknesses as the electrode. Figure 4a shows the cross‐sectional HRTEM images of the MoS_2_‐based transistors with the evaporated Au thin film as the electrode. The right section shows the zoomed‐in image; clearly, the Au atoms bombarded and penetrated the MoS_2_ layers, thus resulting in the discontinuity and unflattening of the MoS_2_ layers. Figure 4b shows the cross‐sectional HRTEM images of the MoS_2_‐based transistors with the transferred Au electrode. No damage or distortion was observed at the interface of the transferred Au electrode and MoS_2_ layers, as shown in the right section of Figure 4b. To compare the interface of the evaporated Au and transferred Au thin films on the MoS_2_ layers, a 3 nm‐thick Au thin film was directly deposited and transferred onto the MoS_2_ layers to investigate the interface using Raman spectroscopy, as shown in Figure S14 (Supporting Information). The full widths at half‐maximum of the E^1^_2g_ peaks for the bare MoS_2_, evaporated Au/MoS_2_, and transferred Au/MoS_2_ were ≈5.1, ≈7.0, and ≈5.5, respectively. Furthermore, the transferred 3 nm‐thick Au/MoS_2_ (red curve) showed a peak position similar to that of the bare MoS_2_ (blue curve). The results confirmed that the quality of the MoS_2_ layers was retained in the Au thin film to serve as the electrode after the transfer process because of the vdW contact. For the evaporated 3 nm‐thick Au/MoS_2_ (light blue curve), a red shift in the E^1^_2g_ peak was indicated (as indicated by the pink arrow), which is typically related to the strain effect.^[^ ^28^ ^]^ Additionally, a broad E^1^_2g_ peak was indicated in the evaporated 3 nm‐thick Au/MoS_2_, thus suggesting that the MoS_2_ layers deteriorated during the deposition of the Au thin film.^[^ ^28^ ^]^ Figure 4c shows the measurements of MoS_2_‐based transistors with evaporated Au and transferred Au thin films as the electrode via the transfer length method. Ten devices were measured with no gate voltage (*V*_G_) at five different channel lengths of 5, 10, 15, 20, and 25 μm. Based on acquisition and extraction, the *R*_c_ of the MoS_2_‐based transistors with the transferred Au thin film as the electrode was 8.4 kΩ, which was lower than that of the transistor with an evaporated Au thin film as the electrode (*R*_c_  = 75.4 kΩ). Figure 4d shows the transfer curves of the MoS_2_‐based transistors with evaporated Au and transferred Au thin films as the electrode, with *V*_G_ biases swept from –10 to 40 V at a drain voltage (*V*_D_) of 1.1 V. Clearly, the *I*_on_ of the transferred Au/MoS_2_‐based transistor was higher than that of the evaporated Au/MoS_2_‐based transistor. Moreover, the SS was lower (i.e., a steep slope before the transistor was turned on) for the transferred Au/MoS_2_‐based transistor. Figure 4e,f shows the output curves of the MoS_2_‐based transistors with evaporated Au and transferred Au thin films as the electrodes, respectively. The devices were subjected to a *V*_D_ from 0 to 7 V, with the *V*_G_ varying from 0 to 35 V. Clearly, the output current can be improved by ≈10× for the transistors with the transferred Au thin film as the electrode. The statistical results obtained from 30 devices for each condition are summarized in Figure 4g,h,i. Figure 4g shows a comparison of the mobilities under the two conditions. The mobilities of the transistors with the transferred Au thin film as the electrode reached approximately ≈15 cm^2^ V^−1^ s^−1^ and were generally distributed in the range of ≈7–10 cm^2^ V^−1^ s^−1^ whereas those of the transistors with the evaporated Au thin film as the electrode were generally distributed in the range of ≈1 to ≈5 cm^2^ V^−1^ s^−1^. As shown in Figure 4h, the transistors with the transferred Au thin film as the electrode exhibited a higher normalized *I*_on_ by 5× to 10× compared with that of the transistors with the evaporated Au thin film as the electrode. Regarding the on/off ratio, the transistors with the transferred and evaporated Au thin films as the electrode achieved average on/off ratios exceeding 10^5^ and less than 10^5^, respectively.

**Figure 5:**
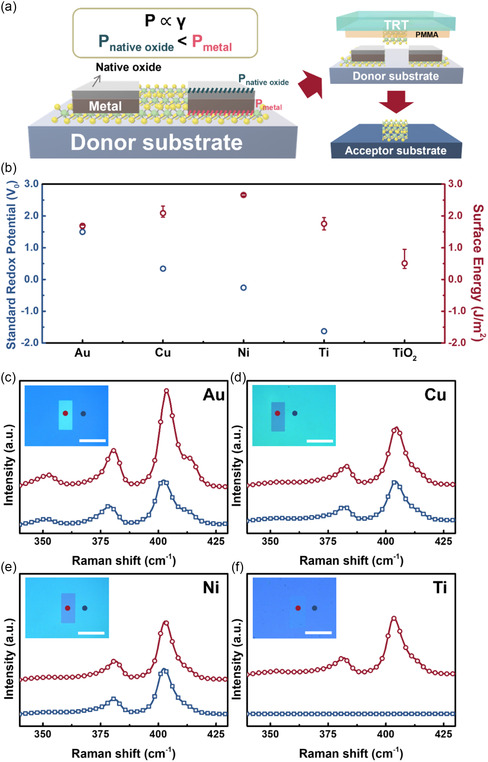
Selective transfer by the TRT/PMMA bi‐supporting layers. a) Schematic illustration of the concept of selective transfer. b) The standard redox potential and surface energy as a function of different materials. c–f) Raman spectra inside and outside the patterns of the MoS_2_ with Au (c), Cu (d), Ni (e), and Ti (f) barriers. The scale bars of the OM image inserts in (c)–(f) are 100 μm.

To augment the functionality of this versatile top‐down transfer process using TRT/PMMA bi‐supporting layers, a metal layer was deposited on the TMDC layers to serve as a barrier for realizing selective transfer, as shown in **Figure** **5**. Figure 5a illustrates the concept of selective transfer based on TRT/PMMA bi‐supporting layers. To realize this concept, using an asymmetric barrier, namely, a metal with a native oxide, can result in different adhesion forces (*P*). Briefly, when the adhesion force between the interface of the PMMA and native oxide (*P*_native-oxide_) is less than that between the interface of the metal and TMDC layers (*P*_metal_), the metal barrier prevents the TMDC layers from being peeled off by the TRT/PMMA supporting layers. Consequently, the TMDC layers uncovered by the metal barrier can be selectively transferred and attached to the acceptor substrate by the TRT/PMMA bi‐supporting layers. Therefore, selecting an appropriate metal barrier is essential for selective transfer processes. Figure 5b shows the standard redox potential (*V*_0_) and surface energy (*γ*) as a function of different materials (Au, Cu, Ni, Ti, and TiO_2_), for which the *V*_0_ of Au, Cu, Ni, and Ti corresponded to 1.498, 0.3419, −0.257, and −1.628, respectively.^[^ ^29^ ^]^ A lower *V*_0_ signifies that the material is more easily oxidized. Notably, the *γ* values of Au, Cu, Ni, Ti, and TiO_2_ were 1.69, 2.09, 2.66, 1.755, and 0.51 J m^−2^, respectively.^[^ ^30^, ^31^, ^32^ ^]^ According to the Derjaguin, Muller, and Toporov theory, *P* is proportional to *γ*.^[^ ^33^ ^]^ Therefore, one can infer that the adhesion force of the Ti thin film with MoS_2_ layers is stronger than that of the TiO_2_ coated with the Ti film containing PMMA. Therefore, Ti with TiO_2_ native oxide can hinder the selective transfer. XPS was conducted to confirm oxidation on the metal surface, as shown in Figure S15 (Supporting Information). Figure S15a–c (Supporting Information) shows the metal core‐level spectra of Au 4f, Cu 2p, and Ni 2p, respectively, which indicate low oxygen concentrations. By contrast, Ti was oxidized easily in the atmosphere and exhibited oxidation‐related features (peaks of Ti^4+^ and Ti^3+^), as shown in Figure S15d (Supporting Information). To confirm the concept of selective transfer processes by TRT/PMMA bi‐supporting layers, Au, Cu, Ni, and Ti thin films were deposited on the MoS_2_ layers as barriers, followed by top‐down transfer by TRT/PMMA bi‐supporting layers to investigate the metal selection for the selective transfer processes on the MoS_2_ layers, as shown in Figure 5c–f. Raman analyses confirmed the existence of both the inside and outside patterns using Au, Cu, Ni, and Ti thin films as barriers after the top‐down transfer processes using TRT/PMMA bi‐supporting layers, as shown in Figure 5c–f, respectively. No MoS_2_ signal was observed outside the patterned region after the selective transfer using a Ti thin film as a barrier (Figure 5f), thus confirming the completion of the selective transfer. By contrast, the MoS_2_ signals were retained both inside and outside the patterns, thus indicating that other metals (Au, Cu, and Ni) peeled off along with the MoS_2_ layers to the donor substrate (SiO_2_ film in the current case), as shown in Figure 5c–e. Therefore, Ti with a native oxide region can obstruct undesired regions after the transfer process to achieve a selective transfer process.

**Figure 6:**
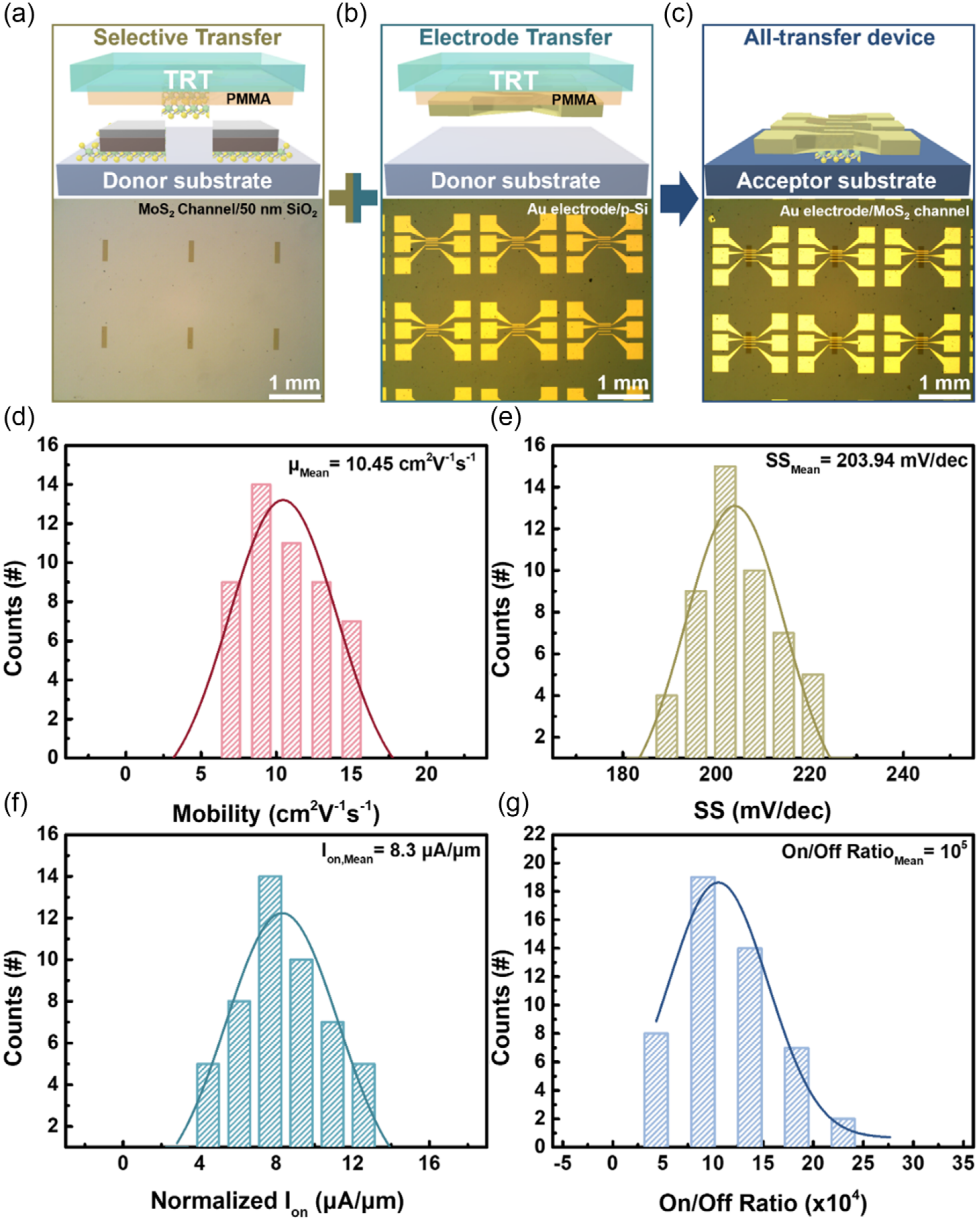
Electrical properties of all‐transferred MoS_2_‐based transistor arrays transferred by TRT/PMMA bi‐supporting layers. a–c) OM images of selective transfer of MoS_2_ layers as channels on SiO_2_/Si substrates (a), Au electrode on p‐Si substrates (b), and all‐transferred devices of transferred Au thin films as the electrode on MoS layers as channels (c). d–g) Histograms of mobility (d), I subthreshold swing (e), normalized *I*_on_ (f), and on/off ratios (g) of all‐transferred MoS_2_‐based transistor arrays.

Eventually, by combining the three functions based on top‐down transfer processes using TRT/PMMA bi‐supporting layers, all‐transfer MoS_2_‐based transistor arrays can be achieved. The overall process for each step is shown in **Figure** **6**a–c. The MoS_2_ layers were selectively transferred as channels onto 50 nm SiO_2_/p^+^‐Si substrates (Figure 6a). Subsequently, the evaporated Au thin film as the electrode was deposited on a p‐Si substrate, followed by the transfer of these Au electrodes onto MoS_2_ layers as channels using TRT/PMMA bi‐supporting layers to form all‐transfer MoS_2_‐based transistor arrays (Figure 6c). Figure S16 (Supporting Information) shows the transfer and output curves of a MoS_2_ transistor from the transistor arrays. The transfer curve exhibited n‐type characteristics, and the output curves exhibited linear behavior, thus demonstrating favorable Ohmic contact. Based on measurements performed on 50 devices with a channel length of 5 μm in the all‐transfer MoS_2_‐based transistor arrays, the mobility, SS, normalized *I*_on_, and on/off ratio were extracted from the transfer curves and then summarized as a histogram, as shown in Figure 6d–g, respectively. Clearly, the mobility was distributed in the range of 8–15 cm^2^ V^−1^ s^−1^, as shown in Figure 6d. Figure 6e shows a histogram of the SS within the range of 190–220 mV dec^−1^. The normalized *I*_on_ ranged from 4 to 14 μA μm^−1^ (Figure 6f) under an on/off ratio ranging between 5 × 10^4^ and 2.5 × 10^5^ (Figure 6g). The statistical results of the field‐effect mobility, SS, normalized *I*_on_, and on/off ratio from the transistor arrays showed uniform distributions with mean values of 10.45 cm^2^ V^−1^ s^−1^, 203.94 mV dec^−1^, 8.3 μA μm^−1^, and 10^5^, respectively. We believe that this study will be beneficial for the development of transfer processes and further applications of TMDC layers‐based devices. **Table** **1** summarizes several transfer technologies for TMDC materials with various characteristics. Radisavljevic et al. used HfO_2_ as the gate oxide to demonstrate a single‐layer MoS_2_ transistor transferred using Scotch tape, and mobility of at least 200 cm^2^ V^−1^ s^−1^ was indicated for this single‐layer MoS_2_ transistor.^[^ ^34^ ^]^ Scotch tape transfer is a straightforward and economical method, thus rendering it suitable for research purposes. This method does not require specialized equipment and is effective for exfoliating thin layers of TMDC materials from bulk crystals. However, this method presents some limitations, including limited control over material placement and orientation, risk of material damage or contamination, difficulties in managing large‐scale or precise transfers, and limited industrial applications. Liu et al. developed a two‐step thermolysis process to synthesize MoS_2_ thin layers that can be easily transferred onto other arbitrary substrates using a PMMA supporting layer. The MoS_2_ showed n‐type behavior with a mobility of 4.7 cm^2^ V^−1^ s^−1^.^[^ ^35^ ^]^ Similarly, we used PMMA in our previous study as the supporting layer with the assistance of a low‐polarity transfer medium to enhance the wettability at the interface between TMDC layers and acceptor substrates. The formation of defects can be reduced on the transferred MoS_2_ flakes, and the MoS_2_ transistor achieved a mobility of 35 cm^2^ V^−1^ s^−1^.^[^ ^10^ ^]^ Bottom‐up transfer (i.e., wet transfer) involving a PMMA supporting layer offers precise control over material placement and alignment, thus rendering it suitable for patterned material deposition using photolithography. However, strain or defects may be introduced into the transferred material, which renders this method unsuitable for large‐scale transfers. Yu et al. demonstrated a 2‐inch MoS_2_ layer transferred using PDMS as a supporting layer, where the MoS_2_ transistor exhibited a mobility of 40 cm^2^ V^−1^ s^−1^.^[^ ^14^ ^]^ The PDMS transfer method offers advantages such as flexibility, thus enabling conformance to substrate contours, particularly delicate or flexible substrates. However, the elasticity of PDMS may result in the twisting and wrinkling of TMDC materials. In addition, the PDMS transfer method involves curing the PDMS mold and is primarily suited for conformal transfers, thus rendering it less ideal for large‐area or patterned transfers. Shim et al. developed a Ni metal film as a supporting layer to achieve the layer transferring of 2‐inch TMDC materials; the transfer of WS_2_ transistors via this method resulted in a high mobility of 89.5 cm^2^ V^−1^ s^−1^.^[^ ^15^ ^]^ Meanwhile, Li et al. demonstrated the bi‐assisted dry transfer of a 2‐inch WS_2_ film and reported that a WS_2_ film with a bi‐metal contact achieved a mobility of ≈30 cm^2^ V^−1^ s^−1^.^[^ ^36^ ^]^ Metal transfer is known for its ability to achieve the precise placement and alignment of TMDC materials, thus rendering it suitable for both large‐area and patterned transfers. However, it presents certain disadvantages, including the necessity for specialized equipment, such as electron beam lithography, higher costs compared with other alternatives, and the potential for metal contamination or adhesion issues that require meticulous consideration during application. Our top‐down transfer method involving a rolling‐assisted TRT/PMMA bi‐supporting layer offers the advantages of highly precise and repeatable transfers, thus rendering it suitable for automated large‐scale manufacturing with minimal contamination or damage risks. However, it requires access to specialized equipment for thermal release, which may incur higher initial investment costs.

**Table 1:** Summary of transfer technologies of TMDC materials with various characteristics

Supporting layer	Transfer method	Growth method of TMDC materials	Transferred film size	Mobility [cm^2^ V^−1^ s^−1^]	Cost‐effectiveness	Industrial scalability	References
Tape	Exfoliation	Bulk MoS_2_	<1 mm	200	Low	Low	[34]
PMMA	Bottom‐up	Thermal decomposition MoS_2_	<1 cm	4.7	Low	Low	[35]
PMMA	Bottom‐up	chemical vapor deposition (CVD) MoS_2_	<1 cm	35	Low	Low	[10]
PDMS	Top‐down	CVD MoS_2_	5 cm (2″)	40	Low	Low	[14]
Metal (Ni)	Top‐down	Vapor phase epitaxy WS_2_	5 cm (2″)	89.5	Medium/High	Medium	[15]
Metal (Bi)	Top‐down	CVD WS_2_	5 cm (2″)	30	Medium/High	Medium	[36]
TRT/PMMA	Top‐down assisted with the rolling process	CVD MoS_2_	5 cm (2″)	10.45	Medium	High	This work

## 3 Conclusion

We have proposed a strategy for a top‐down transfer process (i.e., dry transfer process) to realize large‐scale transfers with fewer transfer‐induced defects on TMDC layers, as well as adopted Au thin films as the electrode based on vdW contacts via TRT/PMMA bi‐supporting layers. The results showed that 2‐inch TMDC layers (MoS_2_ and WS_2_ layers) were successfully transferred onto SiO_2_/Si substrates with high integrity and a high yield of ≈99%. Moreover, the roughness of MoS_2_ layers afforded by the top‐down transfer method using TRT/PMMA bi‐supporting layers was less than 0.6 nm. By applying the adhesion force difference between the metal and metal oxide on MoS_2_ and PMMA surfaces, the selective transfer of MoS_2_ layers was demonstrated, and Ti and TiO_2_ were demonstrated to be the best metal barriers for achieving the best selective transfer results. Additionally, MoS_2_‐based transistors with transferred Au contacts indicated a lower R_c_ and better electrical properties (i.e., mobility, on/off ratio, and *I*_on_) than those based on an evaporated Au thin film as the contact electrode. Furthermore, we demonstrated all‐transfer MoS_2_‐based transistor arrays with selectively transferred MoS_2_ layers as channels and transferred Au thin films as the contact electrode, which yielded uniform electrical properties, i.e., carrier mobility of 10.45 cm^2^ V^−1^ s^−1^, an SS of 203.94 mV dec^−1^, a normalized *I*_on_ of 8.3 μA μm^−1^, and an on/off ratio of 10^5^, respectively.

## 4 Experimental Section

4.1

4.1.1

##### Preparation of TMDC Layers

Large‐scale TMDC layers, such as MoS_2_ and WS_2_, were grown via chemical vapor deposition and metal–organic chemical vapor deposition. Triangular MoS_2_ and monolayer MoS_2_ films were synthesized using MoO_3_ and sulfur powders as precursors, respectively, via chemical vapor deposition.

##### Preparation of Selective Transfer Processes

First, the patterns were fabricated on TMDC layers via photolithography using the AZ5241E photoresist, and the materials (Au, Cu, Ni, and Ti) were deposited via electron beam evaporation as shielding patterns. Subsequently, the substrates were immersed in a PG remover solution at 50 °C for 5 min to remove the photoresist and then rinsed with isopropyl alcohol (IPA) and DI water to ensure cleanliness.

##### Preparation of Au Thin Film as Electrode

The electrode patterns were first prepared on a p‐Si substrate via photolithography using the AZ5241E photoresist, and a 50 nm‐thick Au thin film was deposited via electron beam evaporation as the metal electrode. Subsequently, the metal electrode patterns were immersed in a PG remover solution at 50 °C for 5 min to remove the photoresist and then rinsed with IPA and DI water to ensure cleanliness.

##### Top‐Down Transfer by TRT/PMMA Bi‐Supporting Layers

First, PMMA (Kayaku, PMMA 950 A3) was spin‐coated onto a donor substrate (i.e., the as‐grown TMDC layers, metal pattern/TMDC, and Au electrode) as the first supporting layer at three rotation speeds: 1000 rpm for 10 s, 1500 rpm for 15 s, and 3000 rpm for 30 s. Second, the edges were scraped using tweezers to create a gap between the PMMA/target material (i.e., the TMDC and Au electrodes) and the donor substrate. Subsequently, the PMMA/donor substrate was immersed in a dilute ammonia solution (NH_4_OH:DI water = 1:5) to separate the grown substrate in the subsequent steps. After drying the PMMA/donor substrate with nitrogen gas, a TRT (purchased from Solar Plus company) comprising three layers, i.e., a release liner, foaming adhesive, and PET film, was attached to the PMMA/donor substrate using a rolling machine at a rolling speed of 1 mm s^−1^ and a pressure of 20 kgw as the second supporting layer. Next, the TRT/PMMA/target material was peeled off using DI water and the TRT/PMMA/target material was dried with nitrogen gas. Subsequently, the TRT/PMMA/target material was transferred onto the acceptor substrate using a rolling machine at a rolling speed of 1 mm s^−1^ and a pressure of 20 kgw. The TRT/PMMA/target material/acceptor substrate was placed onto a hot plate heated to 95 °C to eliminate the adhesiveness of the TRT. After the removal of the TRT, the PMMA/target material/acceptor substrate was placed in a hot acetone solution at 80 °C for 40 min to dissolve the PMMA. Subsequently, the target material/acceptor substrate was cleaned with IPA and DI water.

##### Fabrication of Back‐Gate Transistor

A 50‐nm SiO_2_/p^+^‐Si substrate was immersed in acetone, IPA, and DI water and ultrasonicated for 20 min, whereas the TMDC layers were transferred to substrates with TRT/PMMA bi‐supporting layers. Subsequently, the electrode patterns were transferred onto TMDC layers/50‐nm SiO_2_/p^+^‐Si substrates using TRT/PMMA bi‐supporting layers. Finally, the device was rinsed with IPA and DI water to ensure cleanliness.

##### Measurements and Characterization

The size, thickness, morphology, and uniformity of the TMDC layers were characterized using OM, HRTEM (JEOL, JEM‐F200), AFM (Bruker, Dimension Icon), and Raman spectroscopy (CL Technology, UniDRON). HRTEM (JEOL, JEM‐F200, 200 kV) was performed at 200 keV to characterize the cross‐sectional images of the transferred and directly deposited materials. The electrical properties of the devices were measured using a semiconductor parameter analyzer (Agilent, B1500A).

## Conflict of Interest

The authors declare no conflict of interest.

## Author Contributions

Y.C.S. and B.K.W. contributed equally to this work. Y.C.S., B.K.W., and Y. L. C. conceived and coordinated the study. Y.C.S., B.K.W., T.S.T., M.L., J.H.C., T.Y.Y., R.H.C., C.T.C., Y.C.H., C.H.L., Y.Q.H., Y.R.P., and Y.J.Y. performed experiments and data analysis. Y.C.S. and B.K.W. constructed the curves and figures. Y.L.C. provided theoretical guidance. All the authors discussed the results and commented on the manuscript. Y.C.S., B.K.W., and Y.L.C. wrote the manuscript with contributions from all coauthors.

## Supporting information

Supplementary Material

## Data Availability

The data that support the findings of this study are available from the corresponding author upon reasonable request.
