# Supplementary material for: Design of Versatile Top‐Down Transfer by Thermal Release Tape/Poly(methyl methacrylate) (TRT/PMMA) Bi‐Supporting Layers Toward All‐Transfer Transition Metal Dichalcogenide Material Based Transistor Arrays
Source: Small Sci. 2023 Dec 21;4(2):2300144. doi: 10.1002/smsc.202300144 (PMC11935217; doi:10.1002/smsc.202300144)
Supplement: Supplementary file 1 — Supplementary Material [file SMSC-4-2300144-s001.pdf]

Supporting Information

Design of Top-Down Transfer by Versatile TRT/PMMA Bi-Supporting Layers toward All Transferred Transition Metal Dichalcogenide Materials-Based Transistor Arrays

Ying-Chun Shen[a, c, d, †], *Bang-Kai Wu*[a, b, c, d, †], *Tsung-Shun Tsai*[a, c, d], *Mingjin Liu*[a, c, d], *Jyun-Hong Chen*[e], *Tzu-Yi Yang*[a, c, d], *Ruei-Hong Cyu*[a, c, d], *Chieh-Ting Chen*[a, c, d], *Yu-Chieh Hsu*[a, c, d], *Chai-Hung Luo*[a, c, d], *Yu-Qi Huang*[a, c, d], *Yu-Ren Peng*[a, c, d], *Chang-Hong Shen*[e], *Yen-Fu Lin*[f], *Po-Wen Chiu*[b, c], *Ya-Chin King*[b, c*], and *Yu-Lun Chueh*[a, c, d*]

[a]Department of Materials Science and Engineering, National Tsing-Hua University, Hsinchu, 30013, Taiwan

[b]Institute of Electronics Engineering, National Tsing Hua University, Hsinchu 30013, Taiwan

[c]Colleage of Semiconductor Research, National Tsing-Hua University, Hsinchu, 30013, Taiwan

[d]Department of Physics, National Sun Yat-Sen University, Kaohsiung, 80424, Taiwan.

[e]National Applied Research Laboratories, Taiwan Semiconductor Research Institute, Hsinchu, Taiwan

[f]Department of Physics, National Chung Hsing University, Taichung 40227, Taiwan

Hsinchu 30013, Taiwan.

[*]E-mail: ylchueh@mx.nthu.edu.tw and ycking@ee.nthu.edu.tw

[†] Y. C. Shen and B. K. Wu contributed equally to this work.

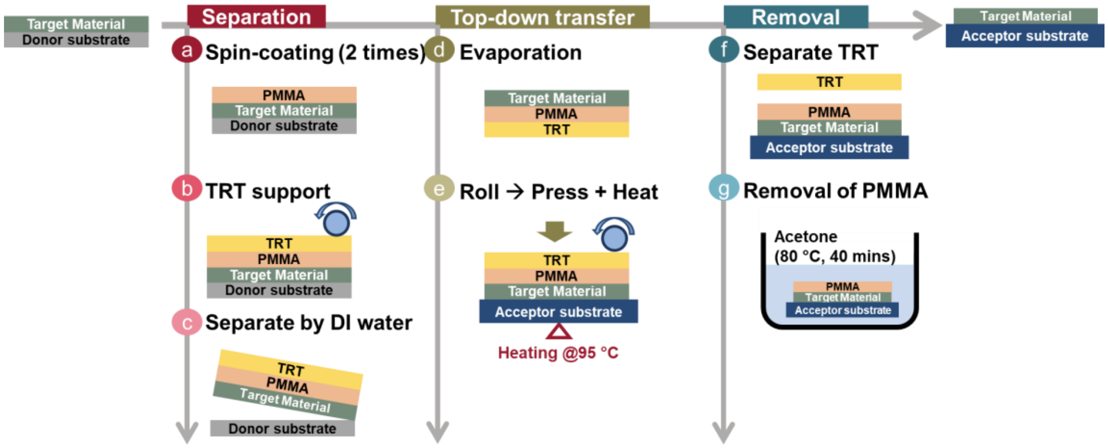

Figure S1 Schematic illustration of the top-down transfer processes using TRT/PMMA bi-supporting layers.

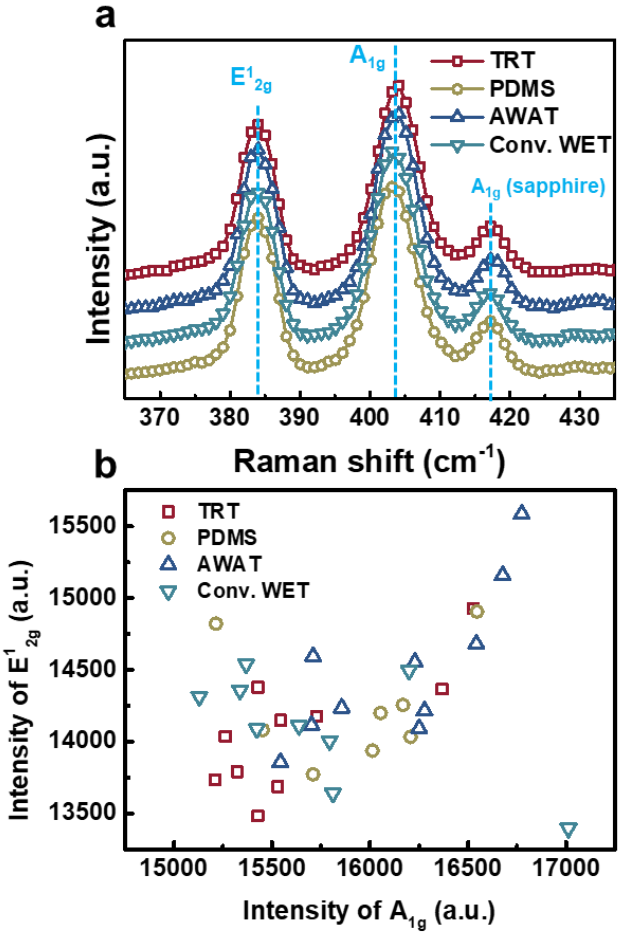

Figure S2 (a) Raman spectra and (b) Statistical intensity results of E^1_{2g} and A_{1g} peaks of MoS_2 flakes after transfer processes by four methods.

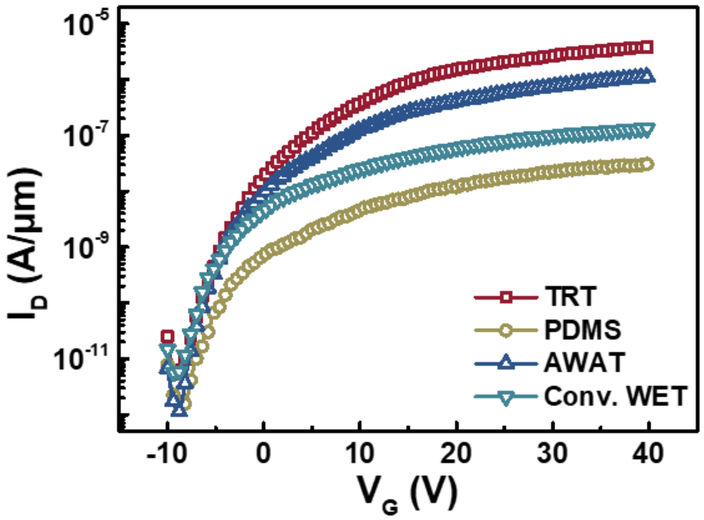

Figure S3 Transfer curves of MoS_2 transistors transfer processes by four methods.

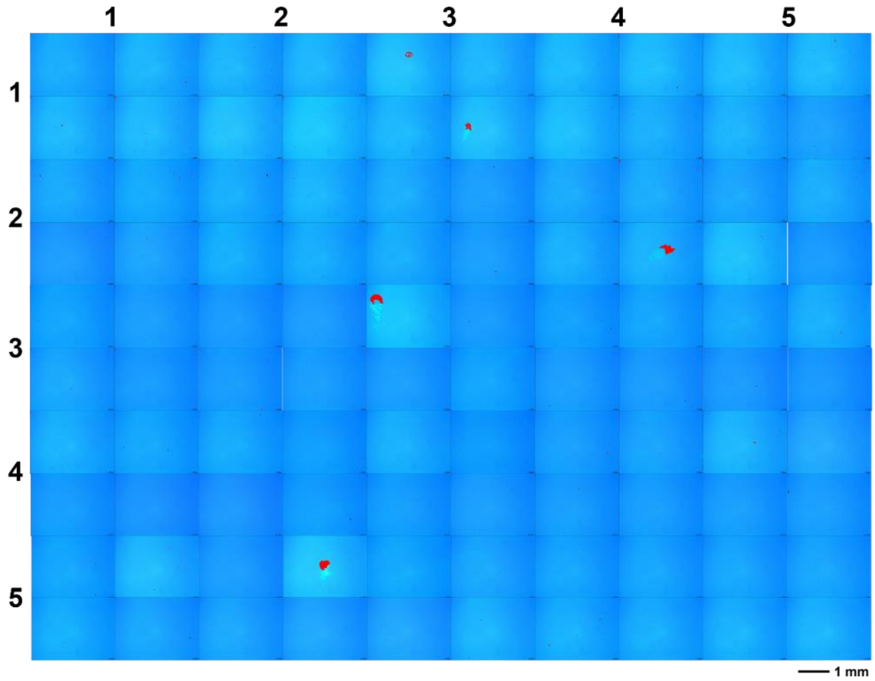

Figure S4 100 OM images collected from a transferred MoS_2/SiO_2 substrate.

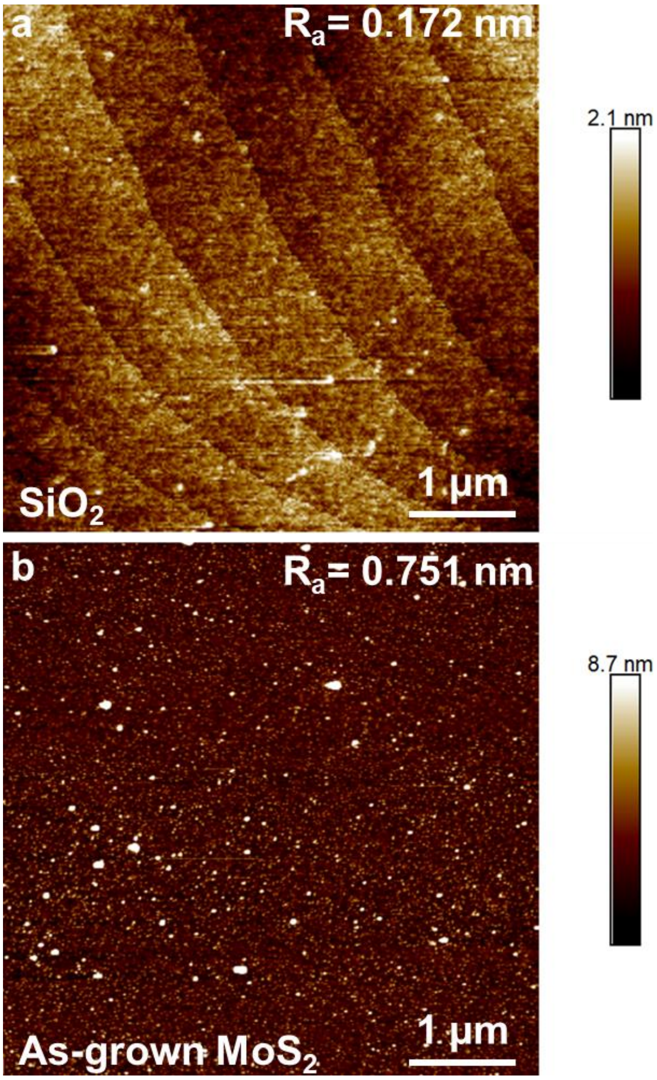

Figure S5 AFM images of (a) SiO_2 substrate and (b) as-grown MoS_2/sapphire.

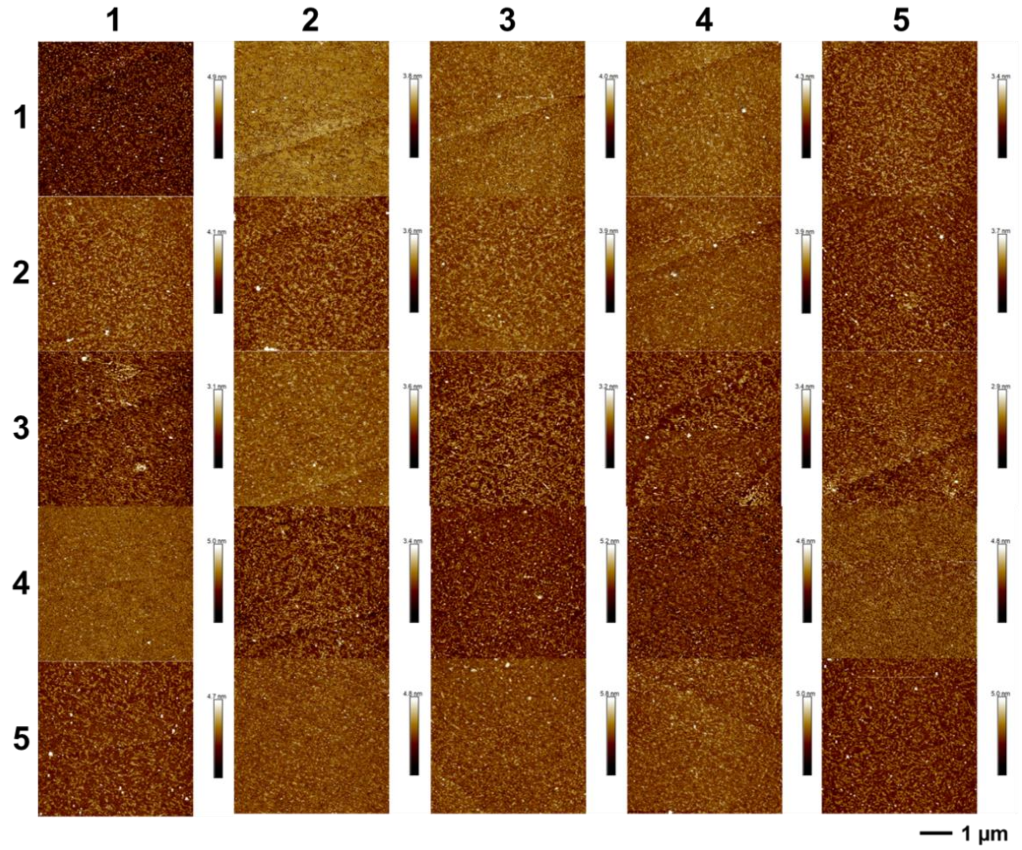

Figure S6 25 AFM images collected from a transferred MoS_2/SiO_2 substrate.

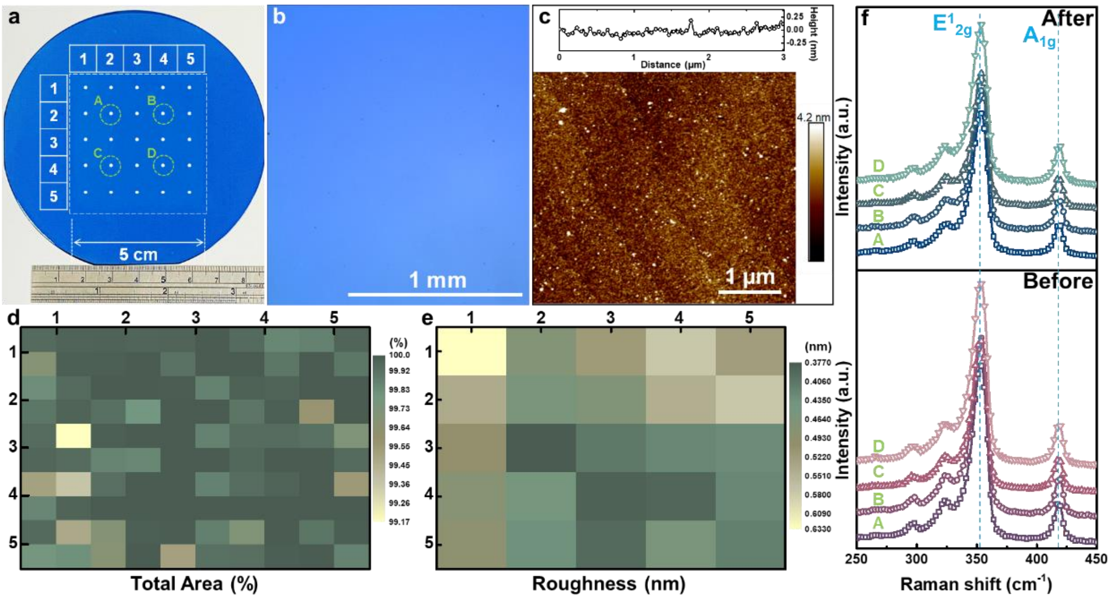

Figure S7 Optical and AFM characterization results of transferred WS_2 layers using TRT/PMMA bi-supporting layers. (a) An optical image of the transferred 5*5 cm^2 WS_2 layers. (b) A OM image, (c) An AFM image, (d) The calculated total transferred area, (e) The roughness distribution, and (f) The Raman spectra before and after transfer of the WS_2 layers.

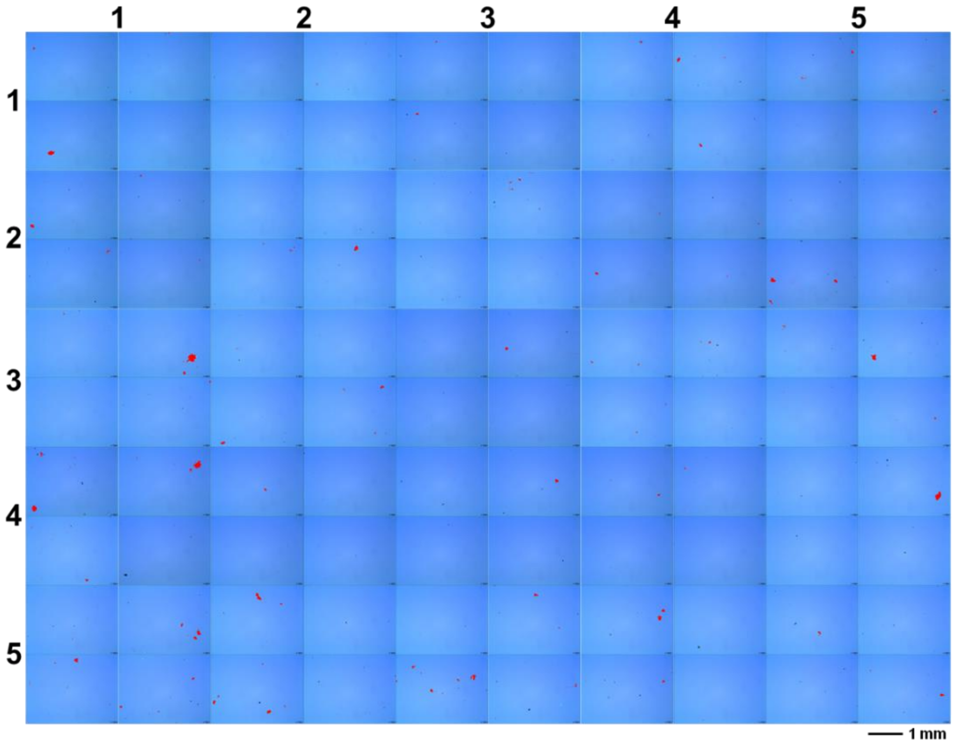

Figure S8 100 OM images collected from a transferred WS_2/SiO_2 substrate.

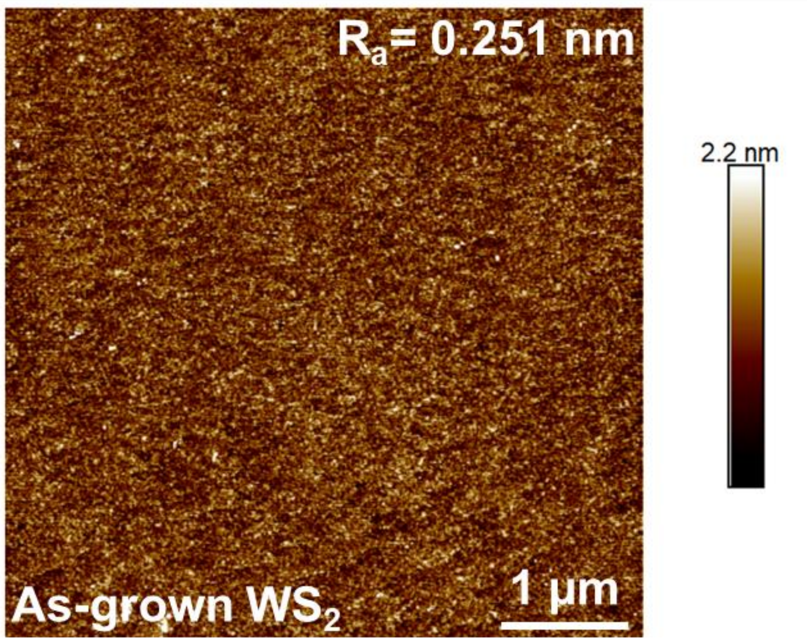

Figure S9 An AFM image of an as-grown WS_2 layers on a SiO_2/Si substrate

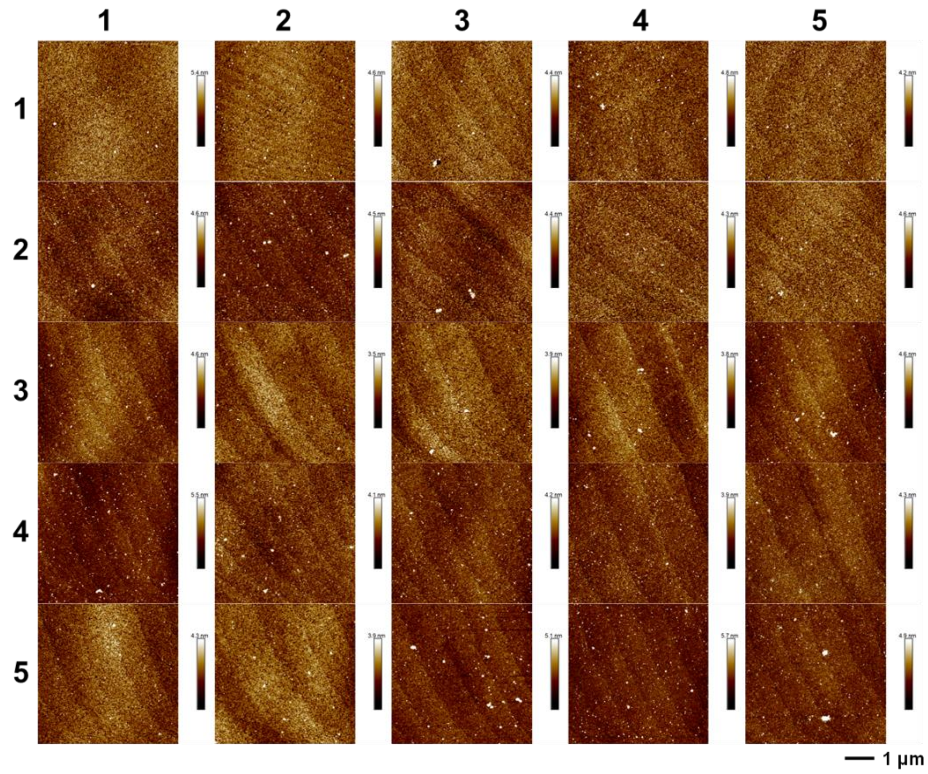

Figure S10 25 AFM images collected from a transferred WS_2/SiO_2 substrate.

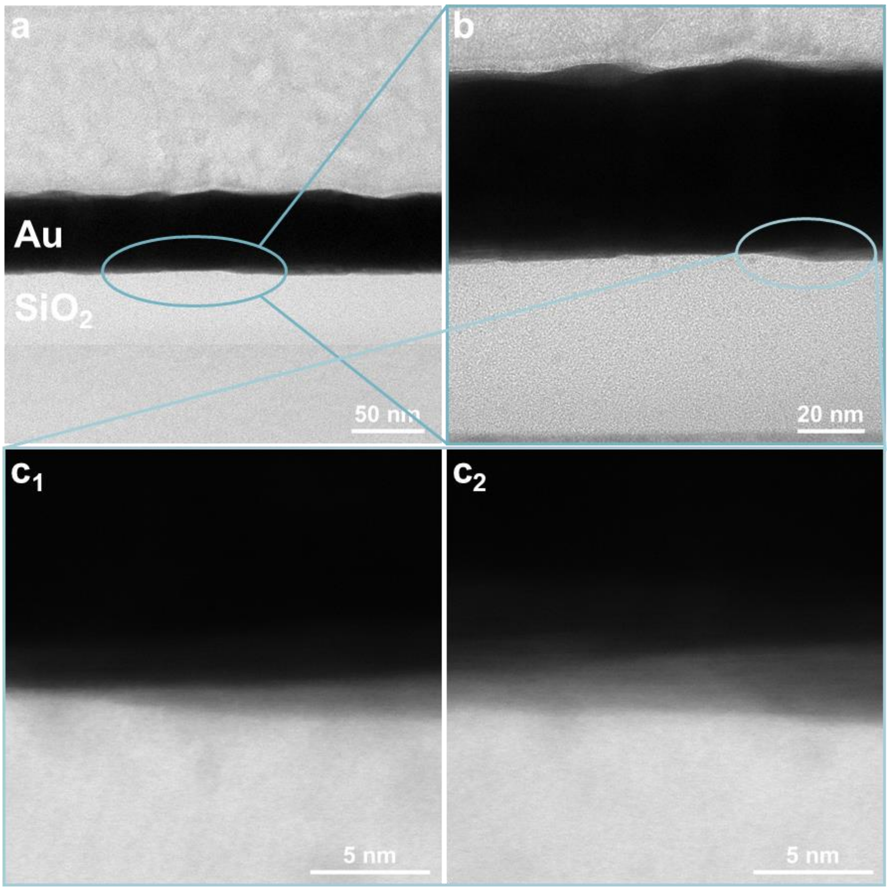

Figure S11 HRTEM images of the evaporated Au thin film on a SiO_2/Si substrate.

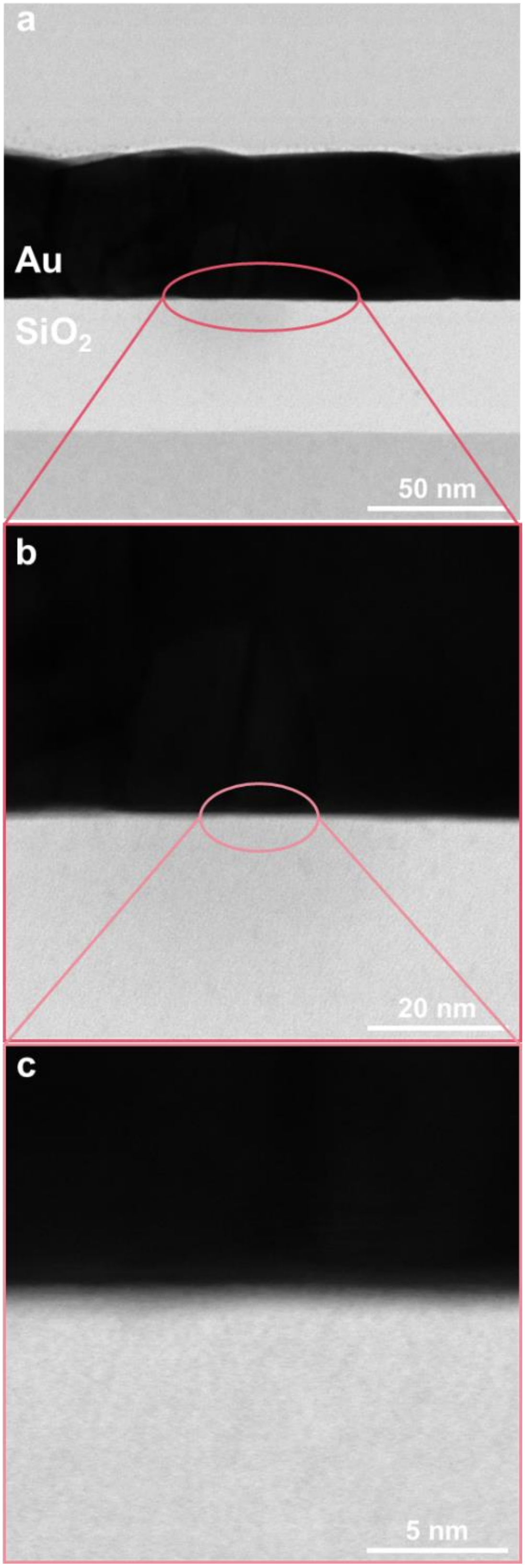

Figure S12 HRTEM images of the transferred Au film on a SiO_2/Si substrate.

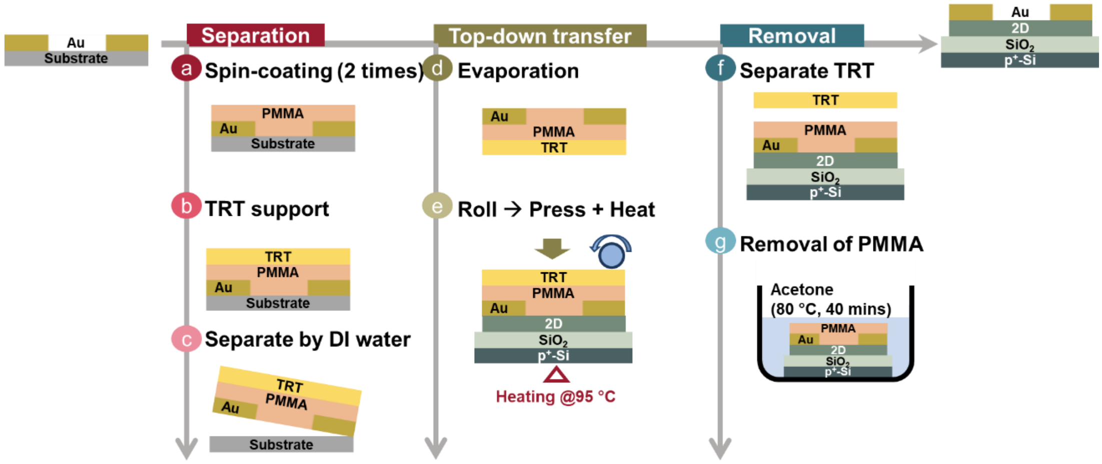

Figure S13 Schematic illustration on the electrode transfer process by the TRT/PMMA bi-supporing layers.

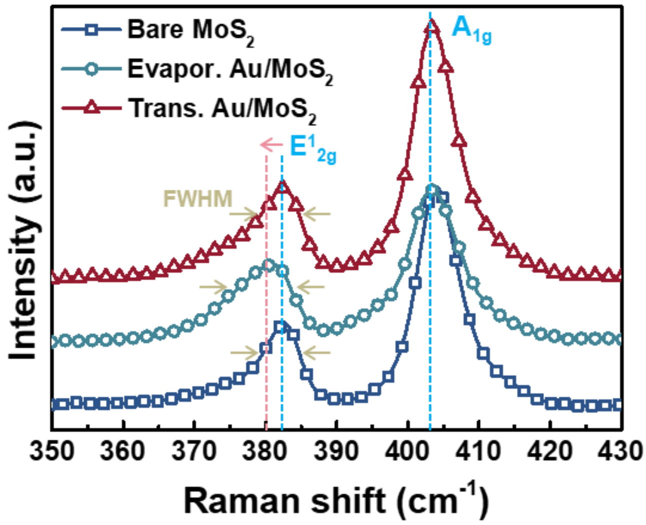

Figure S14 Raman spectra of bare MoS_2, 3 nm-thick evaporated Au/MoS_2, and 3 nm-thick transferred Au/MoS_2.

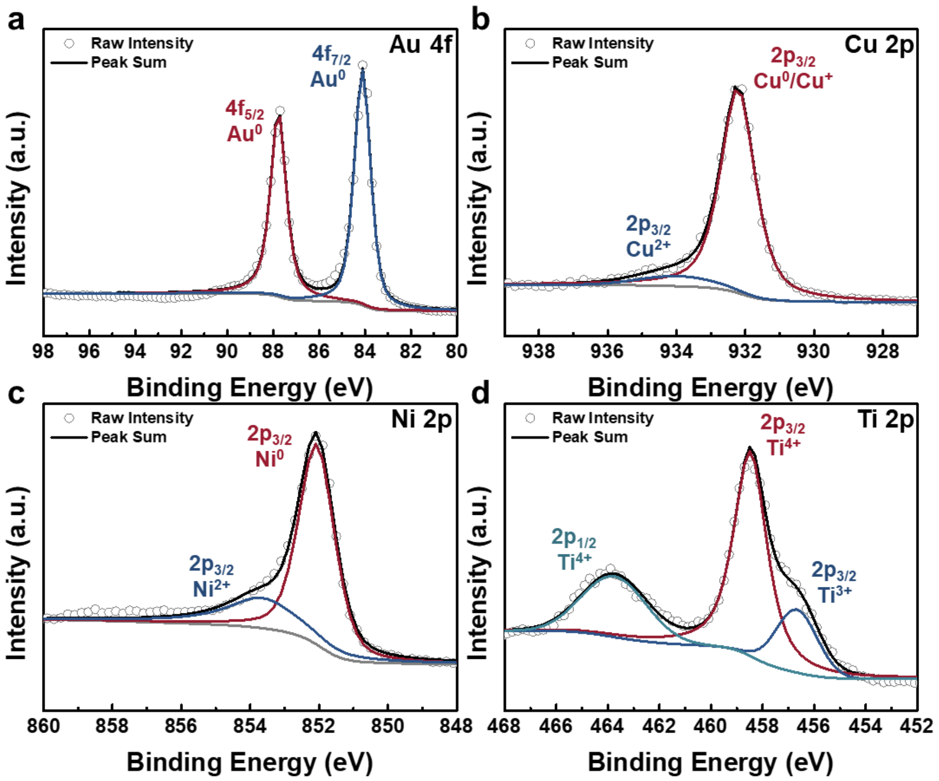

Figure S15 XPS spectra of metal core level of Au 4f, Cu 2p, Ni 2p, and Ti 2p, showing that less oxidation on surfaces of Au, Cu, and Ni metal while the high oxidation on Ti surface can be found.

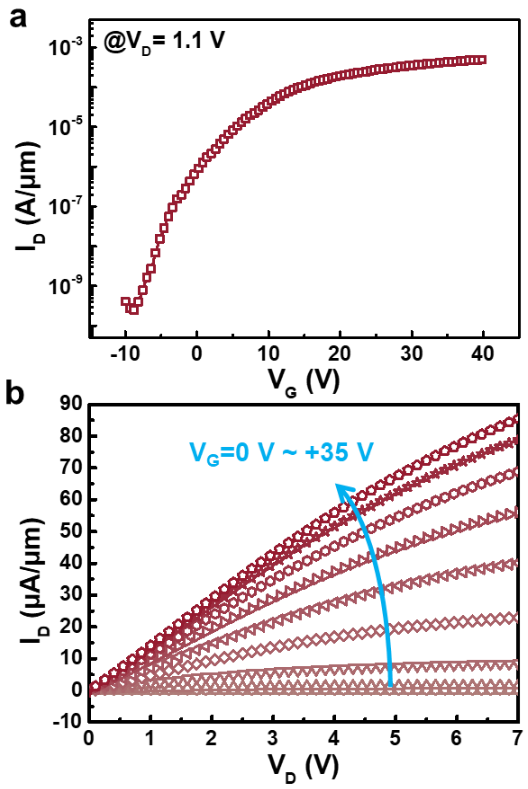

Figure S16 Electrical characteristics of a MoS_2 -transistor from transistor arrays. (a) The transfer curve and (b) the output curves of a MoS_2-based transistor.
